# An Internet-Based Intervention to Promote Alcohol-Related Attitudinal and Behavioral Change Among Adolescents: Protocol of a Cluster Randomized Controlled Trial

**DOI:** 10.2196/resprot.5001

**Published:** 2016-06-01

**Authors:** Patrick Ip, Ko-Ling Chan, Chun-Bong Chow, Tai-Hing Lam, Sai-Yin Ho, Wilfred Hing-Sang Wong, Margaret Fung-Yee Wong

**Affiliations:** ^1^ The University of Hong Kong Department of Paediatrics and Adolescent Medicine Hong Kong China (Hong Kong); ^2^ The University of Hong Kong Department of Social Work & Social Administration Hong Kong China (Hong Kong); ^3^ The University of Hong Kong School of Public Health Hong Kong China (Hong Kong); ^4^ Tung Wah Group of Hospitals Community Services Division Hong Kong China (Hong Kong)

**Keywords:** Internet viral marketing, attitude change, behavioural change, underage drinking, risk behaviour, Internet intervention

## Abstract

**Background:**

Underage drinking is a prevalent risk behavior and common public health problem. Research shows that alcohol abuse not only affects the quality of life of drinkers themselves. The problems resulting from underage drinking pose substantial costs to society as well. The proposed study will address underage drinking with the use of an Internet campaign, which is a cost-effective way of tackling the problem.

**Objective:**

The aims of this study are to test the effectiveness of an online quiz competition in changing adolescents’ alcohol-related attitudes and behavior and to explore the feasibility of using Internet viral marketing to reach a significant number of adolescents.

**Methods:**

The study will constitute a cluster randomized controlled trial for 20 secondary schools (6720 Grade 7-9 students). Schools will be randomized to intervention or control arm with equal likelihood. Students in intervention schools will be invited to take part in the Internet campaign, whereas those in control schools will receive relevant promotional leaflets.

**Results:**

Alcohol-related attitude and behavior will be the primary outcome measures. The results of the proposed study will provide evidence on the efficacy of an Internet intervention in modifying adolescents’ attitudes and behavior and guide further investigation into the prevention of and intervention in such risk behaviors as underage drinking. The project was funded July 2015, enrollment started September 2015, and results are expected July 2017.

**Conclusions:**

With the Internet increasingly being recognized as a practical and cost-effective platform for health information delivery, the proposed Internet-based intervention is expected to be more effective in altering adolescents’ alcohol-related attitudes and behaviors than traditional health promotion.

**ClinicalTrial:**

ClinicalTrials.gov NCT02450344; https://clinicaltrials.gov/ct2/show/NCT02450344 (Archived by WebCite at http://www.webcitation.org/6heB2zMBD)

## Introduction

### Background

Underage drinking is a leading global public health problem. Each year, many underage drinkers die from motor vehicle crashes, homicide, suicide, falls, burns­, and drowning [1]. Research shows that alcohol abuse not only affects the quality of life of drinkers themselves; the problems resulting from underage drinking pose substantial costs to society as well [2]. The proposed study will address underage drinking because, like many other behaviors that pose a health risk such as cigarette smoking and unprotected sex, excessive alcohol consumption habits acquired during adolescence can be tracked into adulthood, thereby harming both current health and health later in life [3]. Studies on the long-term health impacts attributable to alcohol use have found adolescents who start drinking at an early age are more likely to binge drink during adulthood [4]. Moreover, young people’s susceptibility to peer influence peaks during the middle adolescent years, which renders that age group particularly vulnerable to underage alcohol use [5]. In light of these associations and risks, it is recommended that alcohol-related interventions and prevention strategies be initiated as early as during adolescence to obtain maximal benefits.

### Underage Drinking and its Prevalence

Adolescence is a period of stress, during which feelings of insecurity are high and motivation is low [6]. Traditional intervention means may be inadequate to engage youths. As adolescents have been found more open than other age groups to the new possibilities offered by the Internet [7], this medium provides an innovative platform for an intervention program targeting this population. Viral marketing through the peer dissemination of information online may be particularly applicable to changing adolescents’ perceptions and engagement in risk behaviors. The aim of the proposed study is therefore to investigate the extent to which an Internet viral marketing campaign can change drinking behavior and attitudes towards alcohol use among Chinese adolescents.

Underage drinking poses a multitude of risks for teens, their families, and society as a whole, and the prevalence of underage drinking among today’s adolescents is widespread internationally [8]. In 2011, for example, the US National Survey on Drug Use and Health reported that 25% of American youths aged 12-20 drink alcohol, with 16% reporting binge drinking [9]. In Korea, a study of 2124 students attending junior and high schools in Seoul showed that 68% of those aged 12-16 were monthly drinkers and that 28% drank alcohol weekly [10].

The situation in Hong Kong is equally worrying, with the Child Health Survey of 2005/2006 reporting that a significant proportion of children aged 11-14 were current binge drinkers [11]. A more recent survey conducted by the Narcotics Division in 2008 showed that 64.9% of secondary school students had consumed alcohol at least once [12]. The abolition of beer and wine taxes in 2008 and subsequent fierce advertising by the alcohol industry and open endorsement of alcohol by top government officials have further aggravated the local underage drinking problem. School surveys have found a significant increase in monthly alcohol drinking in both adolescent boys (from 19.1% in 2006/7 to 30.4% in 2009/10) and girls (from 16.5% in 2006/7 to 27.5% in 2009/10) after the alcohol tax cut [13]. Hence, there is a pressing need for alcohol-related interventions to stop the growing trend of underage drinking in Hong Kong.

### Transition From Traditional to Internet-Based Alcohol Interventions

Although traditional alcohol interventions by mail or health talks delivered by health care professionals have demonstrated efficacy, it remains difficult to engage adolescents with underage drinking problems who are unwilling or simply do not seek assistance through traditional health services or self-help groups. The Internet, because of its increasing usage among today’s youth, can be an effective medium to engage this high-risk population. There is evidence to support the benefit of online alcohol intervention to reach groups less likely to access traditional alcohol-related services, such as young people, and the evidence also supports its potential to effect behavioral change in large numbers of people [14].

### Internet Viral Marketing and its Efficacy

Internet viral marketing, or the electronic “word-of-mouth” dissemination of information, is one of the best-recognized forms of Internet-based marketing. Research shows that viral marketing is a more effective and efficient marketing tool than traditional media, and one that is characterized by its low cost and exponential transmission of information [15]. A recent prospective pilot study conducted by Ip and Chow assessed the efficacy of an online game-based viral marketing campaign in promoting antismoking attitudes among Chinese adolescents. During the 22-day campaign, the study observed an eightfold increase in the number of participants and a significant attitudinal change, with 73% holding a negative attitude towards smoking after the campaign compared to 57% before it [16]. These promising results suggest the potential effectiveness of adopting a similar viral marketing model to engage youth and promulgate alcohol-related health information among Chinese adolescents.

### Attitudinal Change and its Effects on Behavior

The theory of reasoned action and theory of planned behavior are the most frequently tested models of attitude-behavior relations. Both models view a particular behavior as a function of salient information, or beliefs, relevant to that behavior [17]. Beliefs about the positive outcomes of a behavior lead people to favor and engage in that behavior, while beliefs about its undesirable consequences hinder them from doing so. Research also supports the correlation between attitudinal change and behavioral change [18]. Hence, interventions aimed at changing attitudes towards alcohol use have the potential to induce simultaneous changes in drinking behavior. The aims of this study are to test the effectiveness of an online quiz competition in changing adolescents’ alcohol-related attitudes and behavior and to explore the feasibility of using Internet viral marketing to reach a significant number of adolescents.

## Methods

### Study Design

The proposed study will consist of a cluster randomized controlled trial on the effectiveness of an Internet quiz competition in promoting healthy attitudes and behaviors towards alcohol use among adolescents. As the intervention website could be referred by participants, cluster randomization, instead of individual-level randomization, is chosen to minimize treatment contamination. The study will employ an Internet viral marketing strategy to maximize sample size. In total, 24 schools (6552 Grade 7-9 students) will be recruited with the collaboration of our community partners such as the Tung Wah Group of Hospitals. Considering that the average number of students in one secondary school class is 35 and that there are four classes in each grade, a total of 10,080 students (24×3×4×35) will be reached. Assuming a response rate of 65%, the number of effective campaign starters will be 6552. Students who cannot comprehend basic Chinese and Cantonese will be excluded.

**Figure 1:**
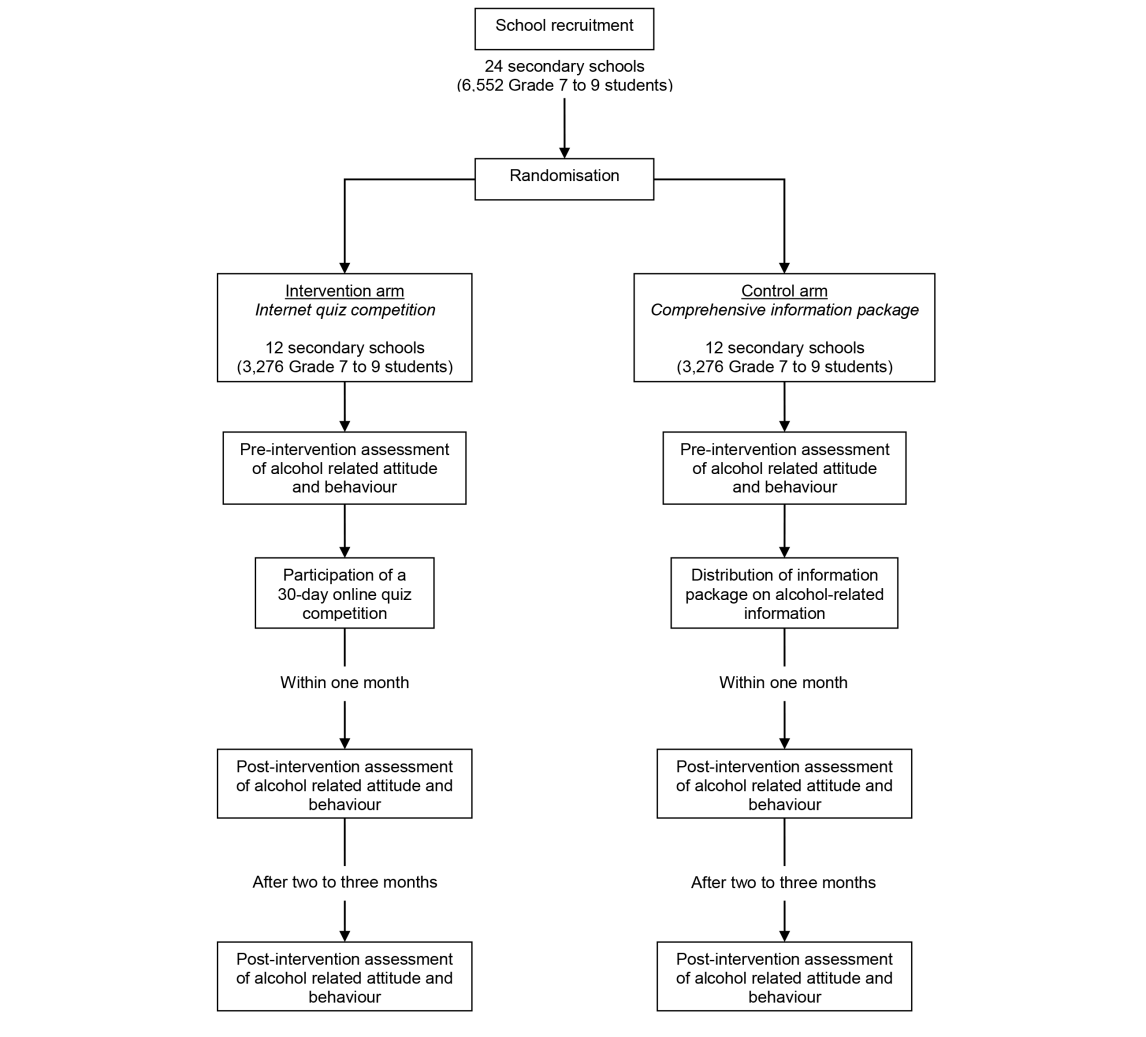
Flowchart of the research design.

Half the schools will be randomized for the Internet quiz competition campaign (intervention arm) and half will be randomized for an information package (control arm). Students in the intervention group will be given the opportunity to take part in the online quiz competition and refer others to join the campaign, whereas those in the control group will receive an electronic information package containing comparable information as the intervention arm. All participating students’ demographics and change in attitude and behavior will be measured by questionnaire surveys before intervention, within 1 month, and between 2-3 months after intervention. A longer-term follow-up (12 months after intervention) will also be attempted if schools are willing to commit and resources allow. Weblog data will also be collected from website servers for subsequent analysis of the effectiveness of the Internet viral marketing strategy. Figure 1 is a flowchart of the proposed study.

### Power Analysis

Considering an attitude difference in 50% of the pooled standard deviation (SD) to be minimally satisfactory, we will need a sample size of 128 to examine, via a two-sample independent *t* test, whether the campaign is more effective than a traditional promotion strategy at a .05 significance level and 80% power [19]. Due to our cluster study design, intraclass correlation (ICC) needs to be addressed. It was reported that an ICC of attitude-related construct among schools was about 0.092 [20], which inflates the required sample size to N=n×[1+(K‒1)×ICC]=128×[1+(3×4×35‒1)×0.092]≈5063. Hence, the number of starters to be recruited (6552) will be sufficient to achieve the study’s primary aim regardless of the number of referrals.

### Intervention Arm: Internet Quiz Competition Campaign

The intervention is designed based on the Theory of Planned Behavior. Participants will gain alcohol-related knowledge through the participation of the intervention, which would in turn alter their attitude concerning drinking and alcohol use. According to the Theory of Planned Behavior, such knowledge and attitude improvement would reduce participants’ intention to drink and thus change or prevent their drinking behavior [21]. To promote the intervention, we will conduct a briefing session and a demonstration session for all eligible students in participating schools in the intervention arm. Campaign promotion brochures and posters will be distributed within those schools.

### Campaign Design

Participants of the campaign will receive a referral code from those who refer them to the online quiz website; starters of the campaigns will receive the code directly from the research team. These referral codes, sets of 8-digit numbers, will be unique to each user and used to track the referral process. During registration, participants will be asked to provide basic demographic information and their referral code. After registration, their baseline attitudes and behavior will be assessed using the Alcohol Attitudes and Behavior survey.

The campaign will last for 30 days, starting from the day of the school promotion session. The aim of the competition from the participants’ perspective will be to obtain the highest score possible to win a prize. They will have two ways of doing so.

The first way will be to answer the online quiz questions. The po­ints gained in this way will constitute the “answer score.” Alcohol-related multiple-choice questions will be presented on the webpage one by one in random non-repeated order. Each correct answer will result in 10 points being awarded to the user’s account. Giving an incorrect answer or skipping a question will not result in any points being deducted. Users will be shown the correct answer immediately after they have chosen their own. The purpose of this design is to promote correct alcohol-related knowledge and attitudes.

**Figure 2:**
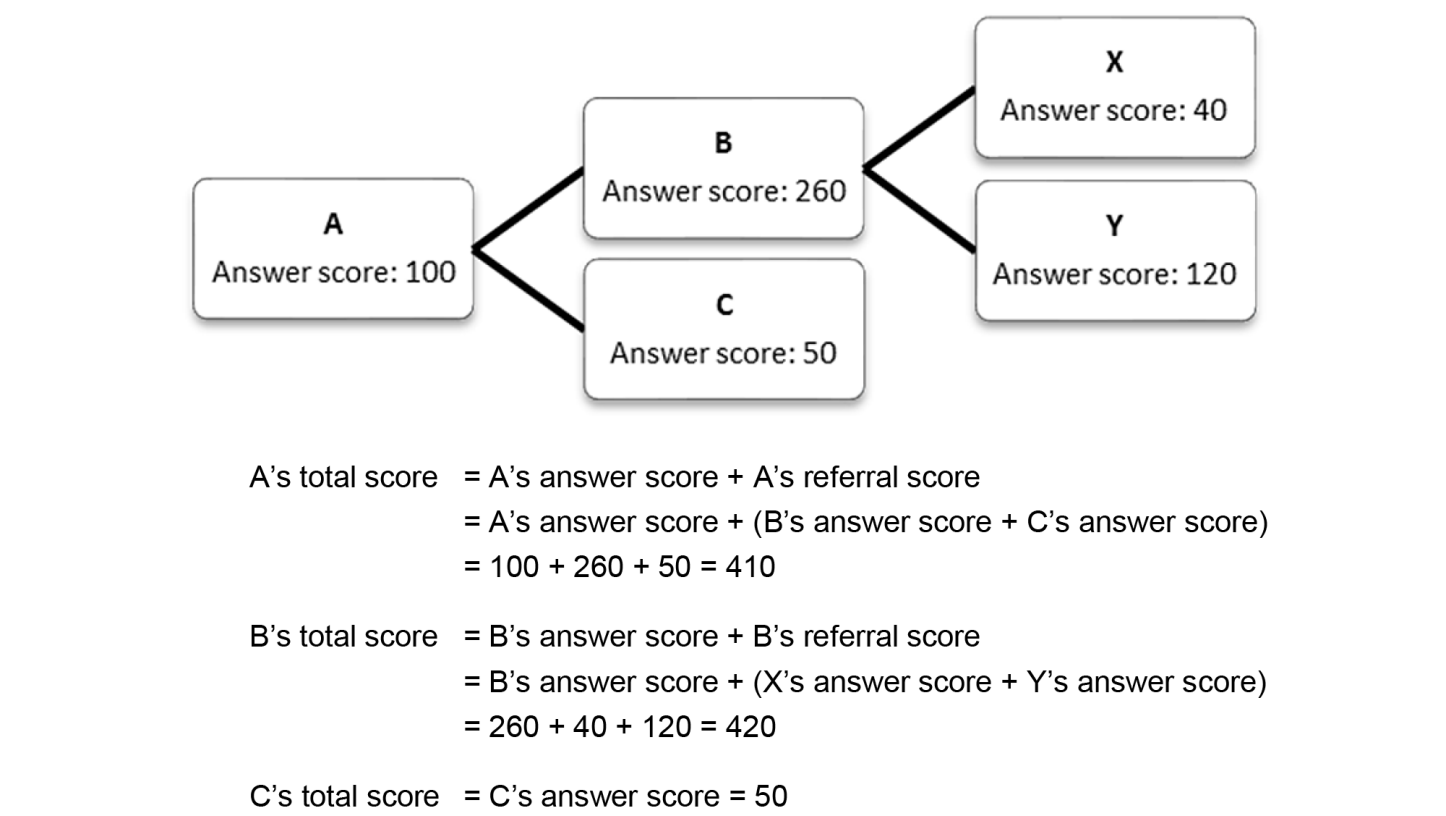
Example of the point system.

The second way of accruing points to obtain a high score will be to make referrals, a step designed to enhance the effectiveness of the Internet viral marketing strategy. Upon a successful referral, the referrer will receive all of the answer scores of their direct referrals (which will constitute their “referral score”). To minimize the chance of self-referrals, users will receive no points from making a successful referral. Furthermore, to ensure campaign competitiveness, the referral scores of lower-level users will not be added to starters’ accounts. The answer score and referral score will be summed into a total score that will be used for prize distribution. Figure 2 illustrates the point system.

### Campaign Website and Quiz Questions

The campaign website will be based on our previous Internet viral marketing campaign, which has shown satisfactory user acceptance [16]. We will make necessary modifications such as enhancing the user interface and preventing duplicate registrations for our study.

Setting the quiz questions will be guided by the Elaboration Likelihood Model (ELM) of persuasion. The quiz questions will be designed by youth volunteers to convey accurate and interesting facts based on international and local sources such as World Health Organization and Hong Kong Department of Health reports and research articles from peer-reviewed journals. The questions will be concrete, specific, and of personal relevance to the participants so that they will be more likely to adopt a “central path” of message processing, which the ELM predicts will induce a more sustainable effect [22]. An experienced clinician and a research assistant will examine every set of 100 questions and discuss them with the question setters to ensure language accuracy and information reliability. This process will be repeated until a total of 1000 quiz questions are generated. Finally, all of the questions will be proofread, refined, and approved by a local alcohol research team from the School of Public Health, University of Hong Kong.

### Incentive Scheme

An incentive scheme will be used to encourage active participation. The 10 highest scorers in each school will receive cash coupons of different values. Table 1 presents the detailed incentive scheme for each school.

**Table 1:** Prizes for the campaign winners in each school.

Rank of total score	Total number of prizes	Prize for each school	Value (HK$)
1	1	Cash coupon	500
2-3	2	Cash coupon	300
4-10	7	Cash coupon	200
Total			2500

### Control Arm: Information Package

A comprehensive package on alcohol-related information will be distributed to all students in the control arm. The information package will contain both printed promotional leaflets from the Department of Health and an electronic guideline for understanding alcohol-related information. The electronic guideline sent through the school intranet system would serve as a unidirectional knowledge transfer medium, which would include a brief summary of the most recent research regarding alcohol and a comprehensive list of relevant website and information sources. We will use these relevant websites and information sources in setting the quiz questions for the intervention arm. As a result, both intervention and control arms could have comparable accessibility to alcohol-related information and the sole contrast between the two groups would be the method of presentation (interactive online quiz competition vs unidirectional information package).

Due to the referral nature of the Internet campaign, it is possible that a small portion of students in the control group school would be referred to participate in the quiz competition, which could in turn dilute the effect size estimate. As a result, we will ask the students in the control group in the postintervention survey whether they have participated in the Internet quiz competition campaign and exclude those who participate in subsequent data analysis.

### Outcome Measure

#### Alcohol Use and Experience

Alcohol use and experience will be evaluated using the 17 items in the Alcohol Attitudes and Behavior survey before and after the intervention. These items are adapted from the Centers for Disease Control and Prevention’s Behavioral Risk Factor Surveillance System Questionnaire and Global School-based Student Health Survey [23,24]. Alcohol use will be measured by an item on the 30-day recall dosage of alcohol consumption. Reasons for drinking, experience in drinking, and other relevant information will be surveyed with the rest of the 16 items. These items have been used in a national study in China [23,24].

#### Attitude Toward Alcohol Use

Attitude towards alcohol use will also be measured with the Alcohol Attitudes and Behavior survey, which includes 15 items adapted from the Scale for the Measurement of Attitudes Toward Alcohol designed to assess that attitude in three domains: sociability, personal unease, and economic aspects [25]. The scale has been validated among adolescents and has demonstrated good internal consistency (Cronbach alphas ranging from .79 to .91) and valid factor structures. Both domain-specific scores and the total score will be used in analysis. We have already gone through a standard procedure for piloting the questionnaire. To ensure the validity of the survey among Chinese adolescents, we have gone through a standard piloting procedure [26]. Briefly, we have invited professional Chinese translators with experience in public health to translate the questionnaire into traditional Chinese, which was then examined and back-translated into English by the research team. The back-translated version of the questionnaire was checked by the research team again to ensure the language accuracy. The Chinese version of the questionnaire was reviewed by a local expert panel using the Delphi process. The expert team included pediatricians, public health experts, parents of adolescents, and secondary school teachers. After reaching consensus with the expert team, the questionnaire was piloted to adolescents to fine-tune the wording and presentation.

#### Number of Successful Referrals per Participants

When making a referral, a participant will need to provide their unique referral code. The new participant who receives this code will then input it during registration. Our website server will record all of these unique referral codes, which will allow us to subsequently analyze the referral pathways and calculate the number of successful referrals per participant.

Since the change in attitudes and behaviors could vary with age, the above analysis will also be carried out for each grade separately.

### Covariates

Owing to randomization, the confounding effect should be minimized. However, we will still evaluate school-level covariates for data analysis. The covariates we will collect include the number of health education lessons related to alcohol, the school neighborhood socioeconomic factors as extracted from 2011 Hong Kong Population Census, and the school academic quality as operationalized by the university admission rate for each school.

## Results

The project was funded by the Health and Medical Research Fund of the Food and Health Bureau, Hong Kong government, in July 2015. Enrollment started September 2015, and results are expected by July 2017.

## Discussion

### Principal Considerations

Alcohol consumption is prevalent among adolescents in Hong Kong. The prevalence of underage drinking is rising globally, according to the World Health Organization. The rise of underage drinking in Hong Kong has been particularly alarming since the alcohol duty reduction in 2008. It is therefore of the utmost importance that children and adolescents be informed of the harmful effects of excessive alcohol use, particularly at a young age.

The aim of the proposed study is to find a cost-effective way of tackling the problem. With the Internet increasingly being recognized as a practical and cost-effective platform for health information delivery, we expect our Internet-based intervention to significantly alter adolescents’ attitudes towards alcohol. It is important to provide accurate alcohol-related knowledge early in adolescence. Research shows the correlation between attitudinal and behavioral changes. Promoting better attitudes toward alcohol use among adolescents may lead to changes in drinking behavior, thereby mitigating the local underage drinking problem.

### Conclusion

The local resources available for school-based alcohol education are relatively minimal. The proposed study will offer an innovative, cost-effective method of raising adolescents’ awareness of the harmful effects of excessive alcohol assumption. The project will be carried out in collaboration with an academic institution and a non-government organization and will involve shared resources, the exchange of best practices, and joint efforts towards sustainability. In the long term, the aim is for the online alcohol education platform to be funded on an ongoing basis and to be incorporated into Hong Kong’s mainstream secondary school health education curriculum. The platform can also be adapted to deliver health-related information in other learning areas such as physical activities, drug use, and safe sex in the near future.

## Abbreviations

ELM: Elaboration Likelihood Model
ICC: intraclass correlation
